# *Hedyotis diffusa* Combined with *Scutellaria barbata* Are the Core Treatment of Chinese Herbal Medicine Used for Breast Cancer Patients: A Population-Based Study

**DOI:** 10.1155/2014/202378

**Published:** 2014-03-09

**Authors:** Yuan-Chieh Yeh, Hsing-Yu Chen, Sien-Hung Yang, Yi-Hsien Lin, Jen-Hwey Chiu, Yi-Hsuan Lin, Jiun-Liang Chen

**Affiliations:** ^1^Division of Chinese Internal Medicine, Center for Traditional Chinese Medicine, Chang Gung Memorial Hospital, No.123 Dinghu Road, Guishan Township, Taoyuan County 333, Taiwan; ^2^Graduate Institute of Clinical Medical Sciences, College of Medicine, Chang Gung University, Taoyuan County 333, Taiwan; ^3^School of Traditional Chinese Medicine, Chang Gung University, Taoyuan County 333, Taiwan; ^4^Institute of Traditional Medicine, School of Medicine, National Yang-Ming University, Taipei 112, Taiwan; ^5^School of Medicine, National Yang-Ming University, Taipei 112, Taiwan; ^6^Division of Radiotherapy, Cheng-Hsin General Hospital, Taipei 112, Taiwan; ^7^Division of General Surgery, Department of Surgery, Taipei Veterans General Hospital, Taipei 112, Taiwan; ^8^Division of General Surgery, Cheng-Hsin General Hospital, Taipei 112, Taiwan

## Abstract

Traditional Chinese medicine (TCM), which is the most common type of complementary and alternative medicine (CAM) used in Taiwan, is increasingly used to treat patients with breast cancer. However, large-scale studies on the patterns of TCM prescriptions for breast cancer are still lacking. The aim of this study was to determine the core treatment of TCM prescriptions used for breast cancer recorded in the Taiwan National Health Insurance Research Database. TCM visits made for breast cancer in 2008 were identified using ICD-9 codes. The prescriptions obtained at these TCM visits were evaluated using association rule mining to evaluate the combinations of Chinese herbal medicine (CHM) used to treat breast cancer patients. A total of 37,176 prescriptions were made for 4,436 outpatients with breast cancer. Association rule mining and network analysis identified *Hedyotis diffusa* plus *Scutellaria barbata* as the most common duplex medicinal (10.9%) used for the core treatment of breast cancer. *Jia-Wei-Xiao-Yao-San* (19.6%) and *Hedyotis diffusa* (41.9%) were the most commonly prescribed herbal formula (HF) and single herb (SH), respectively. Only 35% of the commonly used CHM had been studied for efficacy. More clinical trials are needed to evaluate the efficacy and safety of these CHM used to treat breast cancer.

## 1. Introduction

Breast cancer is the most common cause of cancer-related mortality among females worldwide and the incidence has been increasing in Asia [1–3]. In Taiwan, breast cancer is the most common female cancer and is the fourth highest cause of death overall in women. The incidence of breast cancer increased 82% from 1995 to 2006, while mortality increased by 14.4% [4, 5]. Although there have been advances in therapeutics for breast cancer, which include trastuzumab and aromatase inhibitors, factors affecting quality of life (QOL), such as pain, fatigue, morbidity due to lymphadenectomy, side effects from chemotherapy or radiotherapy, and menopausal symptoms, are still of considerable concern to patients [6]. Thus management of these discomforts is urgently needed by patients and their clinical importance has been increasing [6].

Complementary and alternative medicine (CAM) has been widely used for breast cancer patients, with up to 70% of these patients requesting CAM [7]. CAM is thought to be beneficial to patients' QOL and only has minimal side effect [8, 9]. In Taiwan, more than 33.3% of breast cancer patients have used TCM at least once, and more than 80% of TCM users have chosen Chinese herbal medicine (CHM) for adjuvant breast cancer therapy [10]. CHM is reported to be effective by 78.7% of the patients as breast cancer therapies that can enhance the immune system, treat cancer, reduce the discomfort of chemotherapy and radiotherapy, and relieve menopausal symptoms [11]. However, large clinical studies examining CHM commonly used for breast cancer have not been performed.

Because CHM prescriptions are usually complicated and network-like prescription patterns based on numerous connections between CHM are commonly seen. Evaluation of the composition of CHM prescriptions for breast cancer is crucial for determining the core CHM treatment [12]. A core treatment is defined as the most commonly used CHM that is combined in a single prescription to form the major fraction of an herbal prescription for a specific disorder [13]; therefore, each individual prescription consists of the core treatment and modifications based on the patient's signs and/or symptoms. The core treatment of a specific disorder can only be identified by analyzing the patterns of CHM combinations [14].

The term “duplex medicinal” refers to the use of two single herbs (SHs) that are used together to treat a specific disease in order to enhance efficacy or minimize adverse effects [15]. Similarly, in Western medicine, the purpose of the concomitant use of two medicines is to provide a synergistic effect or to reduce toxicity.

The aim of the study was to identify the core treatment of CHM used for breast cancer in Taiwan by analyzing a population-based CHM prescription database. Further clinical trials and studies targeting the detailed mechanisms of the identified CHM will be made easier if the results of present study are used as a starting point.

## 2. Materials and Methods

### 2.1. The CHM Prescription Database

National Health Insurance (NHI) covers over 99% of the inhabitants of Taiwan and has reimbursed medical expenses since 1995. During 2010, NHI spent almost 1.3 billion US$ to treat breast cancer [5]. Both western medicine and all TCM treatments, including CHM, acupuncture, traumatology, and manual therapy, are totally reimbursed by the NHI of Taiwan. Only licensed TCM practitioners are allowed to prescribe CHM and receive NHI reimbursement. Each recorded clinic visit is categorized and digitally entered into the National Health Insurance Research Database (NHIRD). The nationwide database is released in electronic form to the public for academic research; the data include diagnoses and prescriptions of western medicine and CHM, all of which are linked to encrypted patient information.

In this study, the NHIRD of Taiwan was used to analyze CHM prescriptions [14, 16–18]. In Taiwan, all CHM provided by outpatient services are covered by NHI, and inpatient care is not involved. The datasets contained the date of visit, medical service facility, specialty, patient gender, date of birth, and three major diagnoses, which were based on the International Classification of Disease, 9th Revision, Clinical Modification (ICD-9) [19].

Our analysis only focused on processed CHM that had been concentrated into powdered compounds. Only complete CHM that are certified according to good manufacturing practice (GMP) standards are reimbursed by NHI. Two types of CHM, herbal formulae (HF) and SH, are reimbursed. An SH is a single medicinal plant, whereas an HF consists of several SHs and is based on specific rules. HF and SH are used by themselves or in combination. TCM practitioners can only add an SH rather than substitute a single item in a specific HF when preparing an individual formula.

### 2.2. Data Extraction

**Figure 1:**
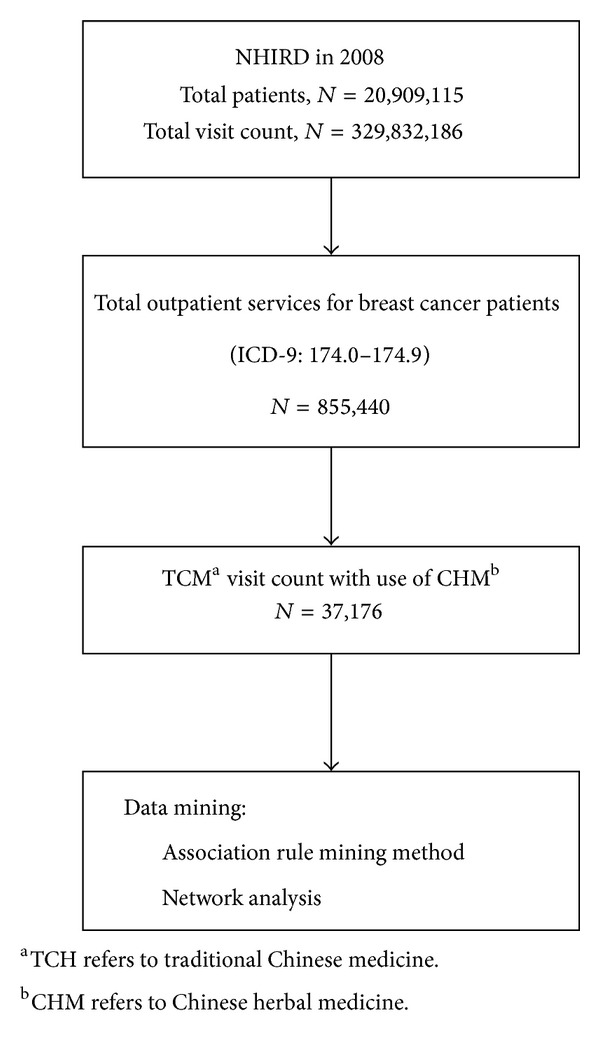
Flowchart of the subject recruitment.

To identify CHM used for breast cancer patients, visits recording at least one diagnosis of breast cancer (ICD-9 codes 174.0–174.9) during 2008/1/1 and 2008/12/31 were extracted from the NHIRD. Among these breast cancer visits, any prescription containing CHM was defined as a TCM visit. The CHM prescriptions for each visit were then extracted to create a prescription database. Visits prescribing acupuncture, massage, or traumatology were excluded in order to avoid possible confounding factors to CHM prescription. The flowchart depicting the selection of patients and data from the NHIRD is shown in Figure 1.

### 2.3. Ethical Approval

The study was approved by the Institutional Review Board of the Chang Gung Memorial Foundation (number 99-3502B).

### 2.4. Data Analysis

The software ASIQ 12.5.7 (Sybase Inc., Dublin, USA) was applied to carry out the data processing. Specifically, association rule mining was used to analyze the prescription patterns. Association rule mining technique was originally designed to find groups or sets of items that are likely to be purchased together in a supermarket and has been applied successfully in determining CHM combination patterns in previous studies [14, 16, 20].

The following two parameters were used for association rule mining: support factors and confidence factors. A support factor refers to the ratio of coprescriptions of drug A plus drug B for all prescriptions, and the confidence factor is the ratio of coprescriptions of drug A plus drug B for all prescriptions including drug A. In brief, the support factor is the prevalence of all CHM among all prescriptions, and the factors were used initially to rule out uncommon CHM. On the other hand, the confidence factors were used to determine the strength of connections between every two CHM combinations and were based on conditional probability. The detailed algorithm was described in our previous work about core prescriptions for pediatric asthma [12]. The support factor threshold was set at 0.5%, which means that only combinations appearing in more than 0.5% of all possible combinations in the prescription database were selected for analysis. Moreover, the threshold of the confidence factor was set at 30% in order to identify statistically significant combinations after iterative trials.

To identify the core treatment of CHM used to treat breast cancer, all the selected combinations were depicted in a network using the freeware NodeXL (http://nodexl.codeplex.com/). Degree was defined as counts of connections to a certain CHM, and higher degrees meant that the CHM was more crucial to form the prescription [21, 22]. Based on this visualized network, the CHM with higher prevalence and degree would be closer to the center of the entire network, especially when only the commonly used CHM were presented in the network. Consequently, the core treatment of CHM can be discovered by examining the central area of the network [12, 23, 24].

## 3. Results

A total of 61,214 beneficiaries' claims in the NHIRD with at least one diagnostic code associated with breast cancer were reimbursed by the NHI in 2008. The total number of outpatient visits associated with the breast-cancer-related beneficiaries' claims was 855,440, and among these, there were 37,176 (4.35%) visits involving 4,436 (7.25%) patients that included a CHM prescription.

### 3.1. Patient Characteristics

The mean age of breast cancer patients using CHM was 52 years. More than 80% of breast cancer patients using CHM were aged between 40 and 59 years (Table 1). The associated symptoms of breast cancer patients using CHM are listed in Table 1. The most common ICD-9 comorbidity of breast cancer patients was ill-defined conditions (478 patients, 10.8%) followed by diseases of the digestive system (446 patients, 10.0%) and diseases of the musculoskeletal system and connective tissue (330, patients, 7.4%).

### 3.2. CHM Prescriptions for Breast Cancer

**Figure 2:**
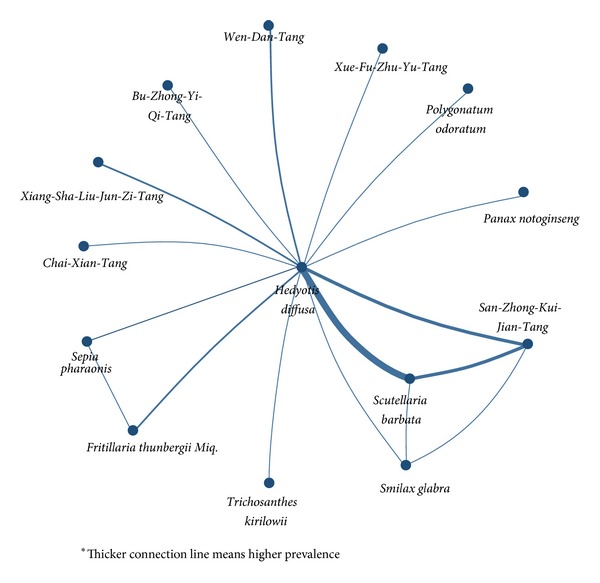
Network of Chinese herbal medicine (CHM) combinations used for breast cancer treatment in Taiwan in 2008.

The mean number of CHM prescriptions per visit was 6.5.* Jia-Wei-Xiao-Yao-San *was the most common HF used among the prescriptions (19.6%) followed by the HFs* San-Zhong-Kui-Jian-Tang *(8.0%) and* Xiang-Sha-Liu-Jun-Zi-Tang *(5.8%) (Table 2). The 3 most common SHs, which accounted for more than 70% of all prescriptions, were* Hedyotis diffusa *(41.9%),* Scutellaria barbata *(17.3%), and* Taraxacum mongolicum *(15.0%) (Table 3). The major role of* Hedyotis diffusa* plus* Scutellaria barbata* is shown in the network of CHM used for breast cancer treatment (Figure 2).

### 3.3. Combinations of CHM

*Hedyotis diffusa* plus* Scutellaria barbata *and* Scutellaria barbata *plus* San-Zhong-Kui-Jian-Tang *were the most common combinations of two HFs used for breast cancer treatment (Table 4) with a prevalence (support factor) of 10.9% and 4.0%, respectively. The respective confidence factors were 77.4% and 50.8%. Among all combinations,* Hedyotis diffusa* plus* Scutellaria barbata *was much more important than other combinations due to the highest confidence and support factor. Additionally, the central role of* Hedyotis diffusa* plus* Scutellaria barbata* was clearly presented in the visualized network of all CHM combinations with markedly high connection counts (Figure 2).

## 4. Discussion

### 4.1. *Hedyotis diffusa* Plus* Scutellaria barbata* Are the Core Treatment of CHM for Breast Cancer

The key finding of this study is that* Hedyotis diffusa* plus* Scutellaria barbata* was the core CHM treatment for breast cancer. Not only are they the two most common SHs used individually (Table 3), but there is also a high prevalence of their use as a duplex medicinal (Table 4), which suggests that they have clinical importance. Moreover, the network of CHM combinations demonstrates that this medicinal duplex had the highest number of connections with other CHM (Figure 2). Previous reports about potential anticancer effects of* Hedyotis diffusa *and* Scutellaria barbata* also prove their important roles in treating cancer [25–27].* Hedyotis diffusa *induces apoptosis in human breast cancer cells and also inhibits tumor angiogenesis [26–28]. In addition,* Scutellaria barbata *is a candidate oral drug that has shown excellent efficacy and safety in early phase clinical trials targeting advanced breast cancer [29].

The TCM perspective is that the combination of* Hedyotis diffusa *and* Scutellaria barbata* may have synergistic activityfor “clearing the heat toxin,” Retention of heat toxin is considered to be a major contributor to the pathogenesis of breast cancer and is the major prescribing target for the TCM practitioner. Moreover, the “heat-toxin clearing effect” is similar to the modern concept of “antitumor effect” [13]. Since some adverse gastrointestinal effects have been reported to be associated with* Scutellaria barbata *monotherapy [36], further study assessing whether use of this duplex medicinal leads to reduced toxicity of* Scutellaria barbata* is warranted.

### 4.2. *Jia-Wei-Xiao-Yao-San* Is an Important Adjuvant for Breast Cancer Patients

*Jia-Wei-Xiao-Yao-San* was frequently used as adjuvant therapy for breast cancer patients. Although* Jia-Wei-Xiao-Yao-San *was the most prevalent HF (19.6%, *n* = 7,303) prescribed for breast cancer patients, even higher than part of the core treatment (*Scutellaria barbata, *17.3% of all prescriptions).* Jia-Wei-Xiao-Yao-San* was seldom significantly linked to other CHM and thus it was not illustrated in the network (Figure 2). Similar adjuvant effect can also be found on biomedical perspectives;* Jia-Wei-Xiao-Yao-San* showed remarkable effects on mood disorder instead of direct anti-cancer effects (Table 5).

High prevalence of mood disorder among breast cancer may be the result of why* Jia-Wei-Xiao-Yao-San* had relative higher prevalence than other CHM. Depression is the most common psychiatric disease associated with breast cancer as high as 46% [41]. Association with depression and anxiety increases with the number of ill-defined syndromes or unexplained symptoms [42], and similar disease pattern was also found in our work; ill-defined symptoms are highly associated with CHM users.

*Jia-Wei-Xiao-Yao-San* was developed to cure anxiety, irritability, depression, and hot flashes in women who were suffering from menstrual disorders, all of which are common adverse effects of breast cancer therapy [6, 33]. To date, this formula is still widely prescribed to treat the menopausal syndrome and functional dyspepsia [14, 34]. Additionally,* Jia-Wei-Xiao-Yao-San *is known to have good patient compliance and safety and is without estrogenic or metabolic effects, which may be of concern if the patient is also receiving conventional hormone therapy [33].

### 4.3. Literature Search for Possible Pharmacological Mechanisms of CHM Used to Treat Breast Cancer

As an external validation to determine the reasons for the high prevalence of the CHM revealed in our study, an extensive literature search was undertaken to explore their possible pharmacological mechanisms. The keywords included the English, Chinese Pinyin, and scientific names for the CHM. The corresponding names in Kampo medicine and Korean medicine were also used. The literature review was obtained from PubMed (latest accession date 2013/10/18) and is summarized in Table 5.

Antidepressant and antitumor activity were the two most common desired pharmacological effects of the CHM that were prescribed to treat breast cancer. Most of the SHs were prescribed for antibreast cancer activity. Seven of the 20 CHM (35%) (including HFs and SHs) most frequently used to treat breast cancer had been studied for their efficacy against breast cancer. However, most were* in vitro* studies.

This study discovered that the CHM that were most frequently used had not been investigated by* in vitro*,* in vivo*, or clinical studies. For instance, of the duplex medicine that was found to be the core treatment, only* Scutellaria barbata *had been studied* in vivo* [36]. This finding illustrates the importance of performing additional clinical trials of* Scutellaria barbata* and the duplex medicinal of* Scutellaria barbata* plus* Hedyotis diffusa*.

### 4.4. Benefits and Drawbacks of This Study

TCM is commonly used to treat breast cancer patients [10]; however, there is little information available on the core CHM treatments prescribed by TCM practitioners. Our analysis also found that each prescription consisted of a mean of 6.5 CHM, which was similar to the results of other studies [14]. The high prevalence of prescriptions did not correspond to efficacy, and high number of CHM in each prescription may also increase risk for toxicity.

Data mining analysis is a method that can identify the patterns of commonly used CHM prescribed for a specific disease. Duplex medicinals seemed to be the most common patterns of combined CHM for breast cancer, since 50% of combinations appearing in the ranking list were an SH plus an SH (Table 4). The network analysis also enabled core treatment discovery. The pattern of coprescription of CHM appears to be characteristic of TCM practitioners, and the identified patterns warrant further investigation, which may lead to clinical trials that evaluate safety and efficacy.

This pharmacoepidemiological study used the NHIRD of Taiwan, which is representative of the general population of Taiwan. There are 23 million people enrolled, most of the inhabitants of Taiwan. Moreover, almost all qualified medical institutions are administrated by the NHI of Taiwan, and all claims from every institution are available in the NHIRD. Therefore, this study does not have the selection bias that affects most hospital-based studies.

Our study has several limitations. First, the NHIRD was established primarily for administrative purposes. Therefore, this database does not have data on cancer staging or laboratory parameters, both of which provide information that is likely to affect a physician's management of a patient. It was thus not feasible to analyze the relationship between treatments and disease outcomes. Second, disease outcomes in relation to CHM and the side effects of CHM were unable to be assessed in this study since only prescriptions were included in this cross-sectional database; however, these pharmacoepidemiological data are crucial for candidate selection of further study about efficacy. Third, only GMP-certified processed CHM are reimbursed by the NHI. Patients who received raw CHM material or so-called folk medicine are not included in our study. Study focused on certificated CHM may minimize the risks of contamination of heavy metal and even adulteration.

## 5. Conclusions

Our study found that the core treatment of CHM used to treat breast cancer in Taiwan was the duplex medicinal* Hedyotis diffusa* plus* Scutellaria barbata*. Data mining can be used to analyze CHM prescription patterns as well as to discover the core treatment of a specific disease. Further empirical scientific investigations on the safety and efficacy of the core treatment used to treat cancer are needed.

## Figures and Tables

**Table 1:** Characteristics of breast cancer patients using Chinese herbal medicine (CHM) in Taiwan in 2008 (total number of patients = 4,436).

Characteristics	ICD-9 codes	Number of patients (%)
Age		
≦40		389 (8.77)
40~60		3146 (70.92)
≧60		901 (20.31)
Associated symptoms		
Symptoms, signs, and ill-defined conditions	780–799	478 (10.78)
Diseases of the digestive system	520–579	446 (10.05)
Diseases of the musculoskeletal system and connective tissue	710–739	330 (7.44)
Diseases of the respiratory system	460–519	268 (6.04)
Diseases of the genitourinary system	580–629	261 (5.88)
Diseases of the circulatory system	390–459	198 (4.46)
Endocrine, nutritional and metabolic diseases, and immunity disorders	240–279	193 (4.35)
Diseases of the skin and subcutaneous tissue	680–709	158 (3.56)
Mental disorders	290–319	154 (3.47)
Diseases of the nervous system and sense organs	320–389	98 (2.21)
Injury and poisoning	800–999	78 (1.76)
Diseases of the blood and blood-forming organs	280–289	64 (1.44)
Infectious and parasitic diseases	001–139	8 (0.18)

**Table 2:** The top ten herbal formula (HF) most used for breast cancer treatment in Taiwan in 2008 (total prescription number, *N* = 37,176).

Rank	HF	Ingredients	Number of prescriptions (%)
1	*Jia-Wei-Xiao-Yao-San *	*Paeonia albiflora, Bupleurum chinense, Atratylodes macrocephala,Wolfiporia extensa, Angelica sinensis, Mentha haplocalyx, Glycyrrhiza uralensis, Zingiber officinale, Paeonia suffruticosa, and Gardenia jasminoides *	7303 (19.64)
2	*San-Zhong-Kui-Jian-Tang *	*Scutellaria baicalensis, Anemarrhena aspodeloidea, Phellodendron amurense, Gentiana scabra, Trichosanthes kirilowii, Platycodon grandiflorus, Ecklonia kurome Okam, Bupleurum chinense, Cimicifuga foetida, Forsythia suspense, Glycyrrhiza uralensis, Sparganium stoloniferum, Curcuma zedoaria, Pueraria lobata, Angelica sinensis, Paeonia albiflora, and Coptis chinensis *	2964 (7.97)
3	*Xiang-Sha-Liu-Jun-Zi-Tang *	*Aucklandia lappa, Amomum villosum, Citrus reticulata, Pinellia ternata, Codonopsis pilosula, Atratylodes macrocephala, Wolfiporia extensa, Glycyrrhiza uralensis, Zingiber officinale, and Ziziphus jujuba Mill. *	2161 (5.81)
4	*Gui-Pi-Tang *	*Codonopsis pilosula, Astragalus membranaceus, Atratylodes macrocephala, Wolfiporia extensa, Ziziphus spinosa, Radix Polygala tenuifolia, Angelicae sinensis, Aucklandia lappa, Glycyrrhiza uralensis, and Dimocarpus longan *	2067 (5.56)
5	*Bu-Zhong-Yi-Qi-Tang *	*Astragalus membranaceus, Glycyrrhiza uralensis, Panax ginseng, Angelica sinensis, Citrus reticulata, Cimicifuga foetida, Bupleurum chinense, and Atratylodes macrocephala *	2066 (5.56)
6	*Zhen-Ren-Huo-Ming-Yin *	*Lonicera japonica, Citrus reticulata, Angelica sinensis, Saposhnikovia divaricata, Angelica dahurica, Glycyrrhiza uralensis, Fritillaria thunbergii Miq., Trichosanthes kirilowii, Boswellia sacra, Commiphora molmol, Gleditsia sinensis, and Manis pentadactyla *	2037 (5.48)
7	*Xue-Fu-Zhu-Yu-Tang *	*Angelica sinensis, Rehmannia glutinosa, Prunus persica, Carthamus tinctorius, Citrus reticulata, Paeonia veitchii, Bupleurum chinense, Glycyrrhiza uralensis, Platycodon grandiflorus, Ligusticum chuanxiong, and Achyranthes bidentata *	1954 (5.26)
8	*Shen-Mai-San *	*Panax ginseng, Ophiopogon japonicus, and Schisandra chinensis *	1870 (5.03)
9	*Suan-Zao-Ren-Tang *	*Ziziphus jujuba var. spinosa, Ligusticum chuanxiong, Anemarrhena aspodeloidea, Glycyrrhiza uralensis, and Wolfiporia extensa *	1824 (4.91)
10	*Zhi-Bai-Di-Huang-Wan *	*Rehmannia glutinosa, Cornus officinalis, Dioscorea opposita, Wolfiporia extensa, Paeonia suffruticosa, Alisma orientale, Anemarrhena aspodeloidea, and Phellodendron amurense *	1610 (4.33)

**Table 3:** The top ten single herbs (SH) most used for breast cancer treatment in Taiwan in 2008 (total prescription number, *N* = 37,176).

Rank	SH	Number of prescriptions (%)
1	*Hedyotis diffusa *	15565 (41.87)
2	*Scutellaria barbata *	6423 (17.28)
3	*Taraxacum mongolicum *	5579 (15.01)
4	*Salvia miltiorrhiza *	4477 (12.04)
5	*Ziziphus spinosa *	4353 (11.71)
6	*Millettia dielsiana *	4305 (11.58)
7	*Astragalus membranaceus *	3801 (10.22)
8	*Scutellaria baicalensis *	3551 (9.55)
9	*Fritillaria thunbergii Miq. *	3536 (9.51)
10	*Rheum palmatum *	3301 (8.88)

**Table 4:** The top ten most common combinations of two Chinese herbal medicine (CHM) used for breast cancer treatment in Taiwan in 2008 (total prescription number, *N* = 37,176; *duplex medicinal, defined as two SHs^c^).

Rank	Combination of two CHM	Support factor (%)	Confidence factor (%)	Number of prescriptions
*1	*Hedyotis diffusa, Scutellaria barbata *	10.88	77.36	4045
2	*Scutellaria barbata, San-Zhong-Kui-Jian-Tang *	4.01	50.78	1492
3	*Hedyotis diffusa, San-Zhong-Kui-Jian-Tang *	3.76	47.52	1396
*4	*Taraxacum mongolicum, Millettia dielsiana *	3.22	33.91	1197
5	*Taraxacum mongolicum, Zhen-Ren-Huo-Ming-Yin *	2.58	50.16	960
*6	*Hedyotis diffusa, Fritillaria thunbergii Miq. *	2.34	31.42	870
*7	*Hedyotis diffusa, Smilax glbra Roxb. *	2.29	76.09	853
*8	*Scutellaria barbata, Smilax glbra Roxb. *	2.06	68.33	766
9	*Hedyotis diffusa, Wen-Dan-Tang *	1.92	49.83	715
10	*Hedyotis diffusa, Xiang-Sha-Liu-Jun-Zi-Tang *	1.82	31.33	677

^c^SH refers to single herbs.

**Table 5:** Possible mechanisms of action of the most common Chinese herbal medicine (CHM) used for breast cancer treatment.

CHM	Possible pharmacologic mechanisms	Reference
HF^d^		
* Jia-Wei-Xiao-Yao-San*	Increases plasma TNF-alpha levels in depressed menopausal patients	[30]
	Antidepressant effects	[31]
	Possible selective estrogen receptor modulator	[32]
	Reliefs of climacteric symptoms in postmenopausal women without changing in estrogen level	[33]
	Adjusts the abnormal gastric motility and gastric myoelectrical activity of patients with functional dyspepsia	[34]
* San-Zhong-Kui-Jian-Tang*	Inhibits the proliferation of human breast cancer	[35]
SH^e^		
* Hedyotis diffusa *	Induces Ca(2+)-mediated apoptosis in human breast cancer cells	[26]
	Induces breast cancer cell apoptosis via mitochondrial pathway	[27]
* Scutellaria barbata *	Induces oxidative stress damage in breast cancer cells	[25]
	Botanical extract (Bezielle, BZL101) used for metastatic breast cancer	[36]
* Salvia miltiorrhiza *	Inhibits growth of breast cancer cell	[37]
	Induces apoptosis of breast cancer cell	[38]
* Scutellaria baicalensis *	Protects against doxorubicin-induced cardiotoxicity	[39]
* Rheum palmatum *	Shows cytotoxicity in both ER-positive and -negative breast cancer cell lines	[40]

^d^HF refers to a herbal formula; ^e^SH refers to a single herb.

## Abbreviations

TCM: Traditional Chinese medicine
CAM: Complementary and alternative medicine
CHM: Chinese herbal medicine
HF: Herbal formula
SH: Single herb
QOL: Quality of life
NHIRD: National Health Insurance Research Database
NHI: National Health Insurance
ICD-9: International Classification of Disease, 9th Revision, Clinical Modification
GMP: Good manufacturing practice.
